# Supplementary material for: Hybrid Models and Biological Model Reduction with PyDSTool
Source: PLoS Comput Biol. 2012 Aug 9;8(8):e1002628. doi: 10.1371/journal.pcbi.1002628 (PMC3415397; doi:10.1371/journal.pcbi.1002628)
Supplement: Text S4 — Complete source code for the PyDSTool package (version 0.88.120504). Includes API documentation and help files linking to web pages. This file is identical to the current public release on Sourceforge.net. (ZIP) [file pcbi.1002628.s004.zip › PyDSTool/html/toc-PyDSTool.parseUtils-module.html]

xml version="1.0" encoding="ascii"?


parseUtils


# Module parseUtils

---

## Classes

auxfnDBclass  
 parserObject  
 symbolMapClass  

## Functions

addArgToCalls  
 ast2shortlist  
 ast2string  

collect\_numbers

convertPowers  
 count\_sep  
 doadd  
 dodiv  
 domul  
 doneg  
 dopower  
 dosub  
 ensurebare  
 ensuredecimalconst  

ensureints

ensureparen  
 ensureparen\_div  
 findEndBrace  
 findNumTailPos  
 isHierarchicalName  
 isIntegerToken  
 isNameToken  
 isNumericToken  
 isToken  

isVectorClause

joinAsStrs  
 joinStrs  
 makeParList  
 mapNames  
 mapPowStr  
 parseMatrixStrToDictStr  
 proper\_match  
 readArgs  
 remove\_indices\_from\_range  
 replaceCallsWithDummies  
 replaceSep  
 replaceSepInv  
 replaceSepList  
 replaceSepListInv  

replaceSepQSpec

replaceSepStr

simplify  
 simplify\_str  
 splitargs  
 splitastLR  
 string2ast  
 sym2name  

temp\_macro\_names

temp\_macro\_names\_inv

toCircumflexSyntax

toDoubleStarSyntax

toPowSyntax  
 trysimple  
 wrapArgInCall  

## Variables

API

API\_class  

Continuous

DO\_DEC

DO\_POW

Discrete

DomainType  
 Inf  

LargestInt32

Macheps  
 NAMESEP  
 NaN  
 ONES  

POW\_STR

TENS

ZEROS  

\_all\_complex

\_all\_float

\_all\_int

\_all\_numpy\_complex

\_all\_numpy\_float

\_all\_numpy\_int

\_classes

\_complex\_types

\_constants

\_float\_types

\_functions

\_indentstr  

\_int\_types

\_num\_equivtype

\_num\_maxmin

\_num\_name2equivtypes

\_num\_name2type

\_num\_type2name

\_num\_types

\_objects

\_pytypefromtype

\_real\_types

\_seq\_types

\_symbconsts

\_symbfuncs

all  

alphabet\_chars\_RE

any  
 argmax  
 argmin  
 array  
 asarray  
 atleast\_1d  
 builtinFnSigInfo  
 builtin\_auxnames  
 clip  
 complex  
 complex128  
 complex192  
 complex64  
 complex\_  
 complexfloating  

convert\_power\_reserved\_keywords

double  
 exp  
 finfo  
 fit\_function  
 float  
 float32  
 float64  
 float96  
 float\_  
 floating  
 greater  
 int  
 int0  
 int16  
 int32  
 int64  
 int8  
 int\_  
 integer  
 intersect  
 isfinite  
 less  
 less\_equal  
 log  
 logical\_or  
 mat  
 minpack  
 name\_chars\_RE  
 ndarray  
 norm  
 npy  

null\_predicate

null\_predicate\_class  
 num\_chars  
 ones  
 os  
 predicate\_op  
 protected\_allnames  
 protected\_auxnamesDB  
 protected\_macronames  
 protected\_mathnames  
 protected\_randomnames  
 protected\_scipynames  
 protected\_specialfns  
 putmask  
 remain  

reserved\_keywords

s

searchsorted  
 shape  
 sign  

specialfns

spy  
 sqrt  
 swapaxes  
 syms  
 sys  
 take  

targetLangs

time  
 types  
 zeros  

---

[hide private]
